# An Inulin-Type Atractylodes Macrocephala Polysaccharide Alleviates Weightlessness-Induced Bone Loss in Association with the Nrf2/HO-1 Pathway

**DOI:** 10.3390/nu18152566

**Published:** 2026-08-06

**Authors:** Jinpeng Wang, Rui Wang, Fan Yang, Wenfeng Wang, Ruifang Shen, Siyu Jiang, Qiao Li, Qiuxin Yan, Yinghui Li, Yan Diao, Lijun Wei

**Affiliations:** 1 School of Life Science and Technology, Harbin Institute of Technology, No. 2 Yi Kuang Street, Harbin 150001, China; 2 Laboratory for Space Environment and Physical Sciences, Harbin Institute of Technology, Harbin 150001, China; 3 State Key Laboratory of Space Medicine, China Astronaut Research and Training Center, Beijing 100094, China; 4 College of Life Science, China West Normal University, Nanchong 637002, China; 5 Collaboration Innovation Center for Tissue Repair Material Engineering Technology, China West Normal University, Nanchong 637002, China

**Keywords:** AMP1-1, WIBL, bone homeostasis, Nrf2, oxidative stress

## Abstract

**Background:** Prolonged exposure to microgravity during spaceflight leads to significant bone loss. The phenomenon of weightlessness-induced bone loss (WIBL) continues to intensify as the flight time increases and this has attracted considerable attention of scholars. **Methods:** In this study, proteomic analysis was performed to identify potential signaling pathways involved in WIBL. Subsequently, the effectiveness of Atractylodes macrocephala polysaccharide (AMP) 1-1 on WIBL was investigated using a rat hindlimb suspension (HLS) model and a co-culture model of osteoblasts and RAW264.7 cell line. **Results:** It was found that AMP1-1 treatment improved bone microarchitecture, reduced the bone transformation rate, and enhanced bone biomechanical properties in the HLS model. In the cell co-culture system, AMP1-1 significantly inhibited osteoclastic differentiation, promoted osteoblastic differentiation, and attenuated osteoblastic apoptosis. The interaction between the nuclear factor erythroid 2-related factor 2 (Nrf2) system and antioxidant enzymes represented a key pathway mediating WIBL. Further investigation confirmed that AMP1-1 facilitated the nuclear translocation of Nrf2, up-regulated heme oxygenase-1 protein expression, and enhanced the transcription of downstream antioxidant enzymes. Consequently, it maintained bone homeostasis and protected against bone loss. **Conclusions:** Our findings demonstrate that AMP1-1 protects against WIBL by modulating the Nrf2 pathway and suppressing oxidative stress, offering a potential therapeutic strategy for mitigating bone loss in weightless environments.

## 1. Introduction

The impact of weightlessness on astronauts has always been a hot issue of concern in the field of space medicine. Current research has shown that the space conditions can lead to a decline in bone formation mediated by osteoblasts and an enhancement of bone resorption by osteoclasts [1]. The bone mass loss of astronauts have been reported to reach to 1.5% per month during spaceflight, which is 10 times the rate of postmenopausal women [2]. It has shown that the bone density increases through running exercises or mechanical stimulation, but it takes a long time and it is difficult to fully recover the bone quality [2]. Therefore, it is extremely urgent to explore the pathological mechanism of weightless-induced bone loss (WIBL) and develop new countermeasures.

Excessive oxidative stress is one of the pathogenic mechanisms involved in WIBL. Under the weightless conditions, the levels of reactive oxygen species (ROS) within cells and the signaling pathways mediating bone resorption are enhanced by oxidative stress [3]. In addition, oxidative stress can trigger inflammatory responses, further aggravate the damage of bone tissue and inhibit the function of osteoblasts [4]. The Kelch-like ECH-associated protein 1 (Keap1)/nuclear factor erythroid 2-related factor 2 (Nrf2) system is a crucial regulatory pathway for cellular antioxidant stress. Under physiological conditions, Nrf2 is anchored in the cytoplasm and combines with Keap1 to form Keap1-Nrf2 heterodimer, an important sensor for oxidative stress [5]. Specifically, oxidative signals can change the conformation of Keap1, prevent the degradation of Nrf2 and translocate it into the nucleus, thereby activating the antioxidant response element [6]. High levels of Nrf2 in the nucleus improve the transcription and production of downstream antioxidant enzymes, such as heme oxygenase-1 (HO-1) and superoxide dismutase (SOD), thereby alleviating oxidative stress [7]. Consequently, targeting the regulation of the Nrf2 pathway and reducing oxidative stress are expected to alleviate WIBL.

*Atractylodes macrocephala* Koidz. is a kind of herbal medicine belonging to the Compositae family [8]. As a type of active constituent, Atractylodes macrocephala polysaccharide (AMP) has the characteristics of small molecular weight and easy absorption into the blood, and has been proven to have obvious antioxidant effects [9]. AMP has been reported to alleviate renal injury by inhibiting oxidative stress [10]. In our previous study, we isolated and purified a neutral polysaccharide fragment from AMP and named it AMP1-1. We have determined its chemical structure to be α-D-Glcp-1 → (2-β-D-Fruf-1)7 and verified its ability to enhance osteogenesis [11]. However, the influence of AMP1-1 on WIBL and its underlying mechanism remains unknown. In the current research, the therapeutic effect of AMP1-1 on WIBL was determined in vivo and in vitro. The molecular mechanism was clarified by determining which AMP1-1 protected against WIBL by regulating the Nrf2 pathway and reducing oxidative stress at the animal and cellular levels. The results provide an experimental basis for the treatment of WIBL.

## 2. Materials and Methods

### 2.1. Materials and Reagents

Osteoblasts were isolated from the calvaria of Sprague-Dawley (SD) rats as mentioned previously [12]. Briefly, calvariae were collected from neonatal SD rats within 48 h after birth. The tissues were cut into 1–2 mm^3^ fragments. The fragments were first digested with 0.25% trypsin–0.02% EDTA (Beyotime, Shanghai, China) for 25 min to remove fibrous tissue. They were then digested with 0.1% collagenase I and 0.05% trypsin–0.004% EDTA (Beyotime, Shanghai, China) for 1 h at 37 °C with shaking. The released cells were collected by centrifugation and resuspended in α-MEM containing 10% FBS (Gbico, Vacaville, CA, USA). Fibroblasts were removed by differential adhesion for 20 min, repeated twice. The osteoblastic phenotype was confirmed by cell morphology and alkaline phosphatase staining. RAW264.7 cell line was supplied by the Shanghai Cell Bank of the Chinese Academy of Sciences (Shanghai, China). Both the isolated primary osteoblasts and RAW264.7 cell line were maintained in α-MEM supplemented with 10% FBS and 1% penicillin–streptomycin (Solarbio, Beijing, China) in a humidified incubator (Thermo Fisher, Waltham, MA, USA) at 37 °C. *Atractylodes macrocephala* Koidz. were purchased from a licensed traditional Chinese medicine pharmacy in Harbin, China and identified by the certificate of supplier (100247570308). The detailed extraction, purification, and structural characterization including monosaccharide composition, molecular weight, and glycosidic linkage analysis of AMP1-1 have been reported in our previous study [11]. In brief, AMP1-1 was isolated from the dried rhizome of *Atractylodes macrocephala* Koidz. The powdered material was extracted with distilled water at a solid-to-liquid ratio of 1:20 (*w*/*v*) using ultrasound-assisted extraction. The extract was centrifuged, decolorized with hydrogen peroxide (Adamas, Shanghai, China), dialyzed against distilled water, and deproteinized using the Sevag method. Crude polysaccharides were then precipitated with ethanol (Adamas, Shanghai, China) and collected by centrifugation. The crude polysaccharide fraction was further purified by DEAE-52 cellulose anion-exchange chromatography, followed by Sephadex G-100 gel-filtration chromatography. The resulting homogeneous polysaccharide fraction was designated AMP1-1. Before administration, the purified AMP1-1 powder was dissolved in distilled water and freshly prepared at the required concentrations. The purity of AMP1-1 was determined to be >98% as assessed by high-performance gel permeation chromatography with refractive index detection. Its identity as a polysaccharide was confirmed by the absence of characteristic signals for proteins and nucleic acids in UV spectroscopy. Its chemical structure was identified as α-D-Glcp-1 → (2-β-D-Fruf-1)7.

### 2.2. Animals and Treatments

Male SD rats with 200 ± 20 g weight were purchased from the Animal Center of Harbin Medical University (Harbin, China). All the experimental procedures were approved by the Committees of Animal Ethics and Experimental Safety of Harbin Institute of Technology (IACUC-2021045). The rats were housed in cages (24 °C, 12 h light and 12 h dark periods) with ad libitum access to food and water. After 7 days of adaptive feeding, 30 rats were randomly separated into 5 experimental groups: the control group; the hindlimb suspension (HLS) group; the HLS group treated with 20 mg kg^−1^·day^−1^ AMP1-1; the HLS group treated with 40 mg·kg^−1^·day^−1^ AMP1-1 and the HLS group treated with 60 mg·kg^−1^·day^−1^ AMP1-1. The doses of AMP1-1 were selected based on a series of preliminary experiments conducted before the formal animal study. Based on the observed biological responses and experimental reproducibility, 20, 40, and 60 mg/kg were selected. Sample size was determined based on data from our preliminary experiments. A random number table generated by Excel was used for animal group allocation. The order of daily administration and sample testing was randomized. Cage positions were rearranged regularly to eliminate light and position confounders. Researchers responsible for animal grouping were blinded to subsequent sample testing and statistical analysis. The researchers responsible for animal grouping were blinded to subsequent sample assessment and statistical analysis. All rats and all collected data were included in the final analysis.

The tails of rats in the HLS groups were hung up according to the reported article [13]. The rats in the hindlimb suspension groups were maintained under tail suspension throughout the 8 week experimental period. The suspension apparatus elevated the hindlimbs while allowing the forelimbs to remain in contact with the cage floor. Therefore, the rats were able to move freely using their forelimbs and had unrestricted access to food and water. The rats were temporarily released from suspension only during daily intragastric administration, which lasted approximately 30 s for each animal. They were returned to the suspension apparatus immediately after administration. Food and water were placed within easy reach, and their availability, together with the general condition of the animals, was checked regularly throughout the experiment. AMP1-1 was administered by intragastric gavage to rats every day. Rats of the control group and HLS group were obtained equal volume of water. All biochemical, imaging, biomechanical, histological, and proteomic assessments were performed at the end of the 8-week experimental period, and no intermediate evaluations were conducted. After 8 weeks, the rats were anesthetized using pentobarbital sodium and the blood was harvested. The serum was collected by centrifuging and stored for the following analysis. The left femurs were immersed in 75% alcohol for Microcomputed Tomography (Micro CT) and three-point bending analysis. The right femurs were fixed in paraformaldehyde for H&E staining. In addition, the randomly selected femurs in the control group and HLS group were applied to the isobaric tags for relative and absolute quantification (iTRAQ) experiments.

### 2.3. Micro CT Analysis

The distal femur was scanned with a Quantum GX2 Micro CT (PerkinElmer, Waltham, MA, USA) according to the guidelines [14]. The three-dimensional (3D) imaging data in all spatial directions was analyzed. The trabecular region was designated using a manually drawn special region with the Analyze 14.0 program (Analyze Direct, Stilwell, KS, USA). The volumes of interest with a thickness of 1 mm (100 continuous slices) in the growth plate was analyzed. The morphological evaluation was surveyed and the parameters such as the bone mineral density (BMD), bone volume fraction (BV/TV), bone surface density (BS/TV), trabecular number (Tb. N), trabecular thickness (Tb. Th) and trabecular separation (Tb. Sp) were analyzed.

### 2.4. iTRAQ Analysis

The method of iTRAQ analysis was followed the previous recommendation [15]. An overview of the analysis was displayed explicitly in Figure S1 which showed the procedure of sample preparation, LC-MS/MS analysis, database search and bioinformatics analysis. According to the report [16], the extracted protein from the femurs of the control group and HLS group were used for LC-MS/MS analysis. The obtained sample was loaded into an analytical column for 90 min [17]. The flow rate and the gradient of solvent were based on a previous report [15]. The proteins were identified and quantified according to peptide fragment mass spectrometry. The data was searched and analyzed by optimization and score (unused protein score > 1.3 means the confidence of proteins identified > 95%). According to the *t*-test, the protein with a *p* value less than 0.05 was considered to have significant changes. Folding changes greater than 1.5 or less than 0.67 are regarded as differential proteins. Furthermore, the information of differential expressed proteins (DEPs) in response to oxidative stress are presented in Table S1.

### 2.5. Serum Analysis

The content of cross-linked carboxyterminal telopeptide of type I collagen (β-CTX), tartrate-resistant acid phosphatase 5b (TRAP 5b), cross-linked carboxyterminal telopeptide of type I collagen (ICTP), procollagen type 1 *N*-terminal propeptide (P1NP) and bone alkaline phosphatase (BALP) were evaluated using the ELISA kits (Nanjing Jiancheng Bioengineering, Nanjing, China).

### 2.6. Three-Point Bending Analysis

The bone biomechanical properties were determined based on previous reports [18]. Briefly, the span of the electronic universal testing machine (Instron 8511, Boston, MA, USA) was set to 16 mm, and the descent speed of the load regulator was set to 0.4 mm/min. The femurs were placed in sequence on the stage at the same position. The middle part of the diaphysis sustained the load until the femoral fracture occurs. Finally, the relevant data was recorded and the corresponding parameters were calculated and presented in Table S2.

### 2.7. H&E Staining

In total, 10% EDTA solution was used to decalcify the harvested femurs for one month. A rotary microtome (Leica RM2255, Frankfur, Germany) was used to section the embedded tissues in paraffin of 4 μm thickness. The bone sections were stained and observed using an inverted microscope (Motic, Xiamen, China) for histological analysis [19]. The trabecular bone area percentage (Tb. Ar, %) were analyzed.

### 2.8. Detection of Enzyme Activity

Samples were tested by their respective kits and the procedure was based on our previous articles [20]. The protein of cells or femurs were quantified according to the operation manual. The enzyme activity of catalase (CAT), SOD, glutathione peroxidase (GSH-Px), and alkaline phosphatase (ALP) were calculated based on the following formula.

The activity of CAT, SOD, GSH-Px, and ALP = Detected activity value/total protein.

### 2.9. Quantificational Real-Time Polymerase Chain Reaction (qRT-PCR) Analysis

The total RNA extraction and the detailed description of qRT-PCR can be referred to in previous protocols [20]. The reverse transcription reaction was manipulated with the PrimeScript RT Reagent Kit (TaKaRa, Dalian, China). The total mRNA was quantified by the TaKaRa SYBR^®^ Premix Ex TaqTM II kit (TaKaRa, Dalian, China) and analyzed using the ABI 7500 sequence detection system (Applied Biosystem, Foster City, CA, USA). The gene expression was calculated through the 2^−ΔΔCt^ method [21]. The PCR primers used in this study are presented in Table S3.

### 2.10. Immunohistochemical (IHC) Staining

The procedures of IHC staining can be referred to in the previous study [22]. Briefly, paraffin-embedded sections were deparaffinized and rehydrated. Antigen retrieval was performed in citrate buffer (pH 6.0) at 95 °C for 12 min. After returning to room temperature, the sections were washed with PBS and treated with 3% hydrogen peroxide for 15 min to block endogenous peroxidase activity. The sections were then blocked with 5% bovine serum albumin for 1 h at room temperature and incubated with the corresponding primary antibodies, followed by the appropriate secondary antibodies. Images were acquired and analyzed using a fluorescence microscope (Olympus IX71, Tokyo, Japan).

### 2.11. Co-Culture System Under the Effect of Simulated Microgravity (SMG)

To better mimic the physiological bone microenvironment, in which osteoblast–osteoclast crosstalk plays a critical role in maintaining bone homeostasis, we employed a co-culture system of primary rat osteoblasts and RAW264.7 cell line in this study. This system allows for the evaluation of AMP1-1’s integrated effects on both bone formation and resorption, as factors secreted by osteoblasts under co-culture conditions can promote the differentiation of RAW264.7 cell line into osteoclasts. The third cell passage (P3) osteoblasts and RAW264.7 cell line were seeded separately onto glass coverslips at a density of 1 × 10^6^ cells per coverslip for each cell type. After overnight culture, the coverslips carrying the two cell types were placed in the same 2D-Rotating Wall Vessel (2D-RWVS, Astronaut Research and Training Center, Beijing, China). The device was used to generate SMG conditions. According to the equipment manual and a previously reported method [20], the samples were rotated around the horizontal axis at 30 rpm. During horizontal rotation, the orientation of the cells and the direction of the gravity vector changed continuously, thereby reducing the net gravitational stimulus perceived by the cells over each rotation cycle. The average centrifugal acceleration was approximately 0.2 g. The cells were exposed to SMG for 72 h and then collected for subsequent experiments. Cells cultured in the device without rotation were used as the static control group. The experimental groups were designed as follows: the control group; the SMG group; the SMG group treated with 30 ng/mL AMP1-1; the SMG group treated with 60 ng/mL AMP1-1 and the SMG group treated with 90 ng/mL AMP1-1. The concentrations of AMP1-1 used in our in vitro experiments were determined based on preliminary dose–response experiments conducted prior to the formal study. In these preliminary experiments, it was observed that 30, 60, and 90 ng/mL AMP1-1 promoted osteoblast proliferation and inhibited osteoclast differentiation under co-culture conditions.

### 2.12. Apoptosis Analysis

Annexin V-FITC/PI staining was operated to examine cell apoptosis by the flow cytometry as previous depicted [23]. After the treatment of SMG, the glass slides were taken out and put into a 6-well plate containing PBS buffer. The osteoblasts were digested by trypsin. After washing for three times, the cells mixed with 5 μL annexin V-FITC and 5 μL PI were analyzed by FACSCalibur flow cytometer (BD Biosciences, Franklin Lakes, NJ, USA) after incubating for 15 min.

### 2.13. Western Blot

The total protein was isolated and blotted to the membranes as previously described [24]. The blocked membranes were hybridized with the corresponding primary antibodies: Nrf2 (Proteintech, Wuhan, China, Cat No. 80593-1-RR, Rabbit, 1:1000, 4 °C, overnight), HO-1 (Proteintech, Wuhan, China, Cat No. 10701-1-AP, Rabbit, 1:1000, 4 °C, overnight), GAPDH (Abclonal, Wuhan, China, Cat No. AC027, Rabbit, 1:1000, 4 °C, overnight), Lamin B1 (Proteintech, Wuhan, China, Cat No. 12987-1-AP, Rabbit, 1:1000, 4 °C, overnight). After washing, the membranes were incubated with the secondary antibodies (Multi-rAb^®^ HRP-Goat Anti-Rabbit Recombinant Secondary Antibody (H + L), Proteintech, Wuhan, China, Cat No. RGAR001, Rabbit, 1:10,000, Room temperature, 1 h). After washing, the images were acquired and analyzed through the Automatic chemiluminescence analysis system (Tanon, Shanghai, China).

### 2.14. Statistical Analysis

GraphPad Prism version 8.0 was used to present the data as the mean ± SDs. Differences among multiple-groups were assessed by One-way ANOVA. Results were considered statistically significant at *p* < 0.05.

## 3. Results

### 3.1. Establishment of the HLS Rat Model and iTRAQ Analysis

**Figure 1:**
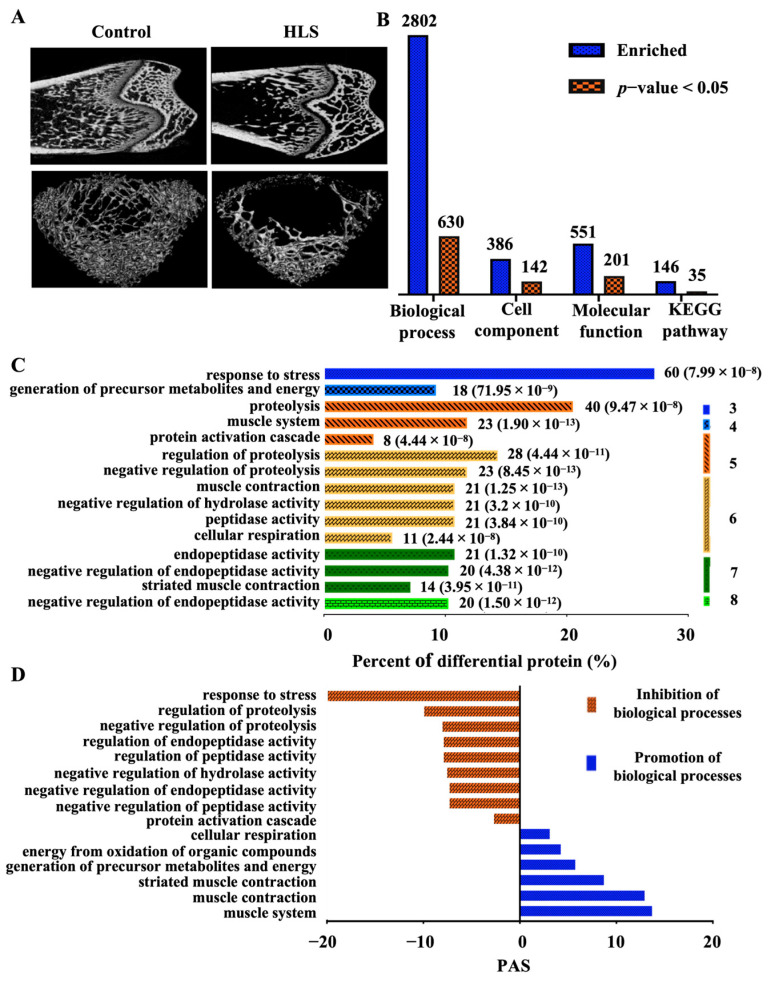
**Response to stress exerted a crucial role in WIBL.** (**A**) Establishment of the HLS rat model (*n* = 6 per group, the statistical results have been presented in Supplementary Figure S2). (**B**) GO enrichment and KEGG pathway analysis of DEPs identified by proteomic analysis. (**C**) Analysis of BP associated with the DEPs. (**D**) Analysis of the effective intensity of DEPs involved in BP. Abbreviations: HLS, hindlimb suspension; GO, Gene Ontology; KEGG, Kyoto Encyclopedia of Genes and Genomes; DEPs, differentially expressed proteins; BP: biological process. DEPs were defined as proteins with fold change ≥1.5 or ≤0.67 and *p* < 0.05.

To establish an animal model of WIBL, rats were randomly divided into the control group and the HLS group. The HLS group received tail suspension for 8 weeks. Micro CT scanning in Figure 1A shows that the HLS group had obvious bone microarchitecture damage compared with the control group (the statistical results have been presented in Figure S2). These results confirmed that the HLS rat model was successfully established. According to iTRAQ analysis in the control and HLS rats, there were a total of 288 DEPs in femurs, among which 163 proteins were up-regulated and 125 proteins were down-regulated. Through Gene ontology (GO) enrichment and Kyoto encyclopedia of genes and genomes (KEGG) analysis, it was found that the number of DEPs enriched in BP, cell component, molecular function and KEGG pathways were 630, 142, 201 and 35, respectively (Figure 1B). The above results indicated that the DEPs in femurs were mainly enriched in BP. A statistical analysis of the top 15 BP in terms of significance revealed that response to stress contained 60 DEPs with a significance of 7.99 × 10^−8^ (Figure 1C). In addition, this study analyzed the effective intensity of DEPs in BP and found that response to stress had the strongest inhibitory effect (Figure 1D).

**Figure 2:**
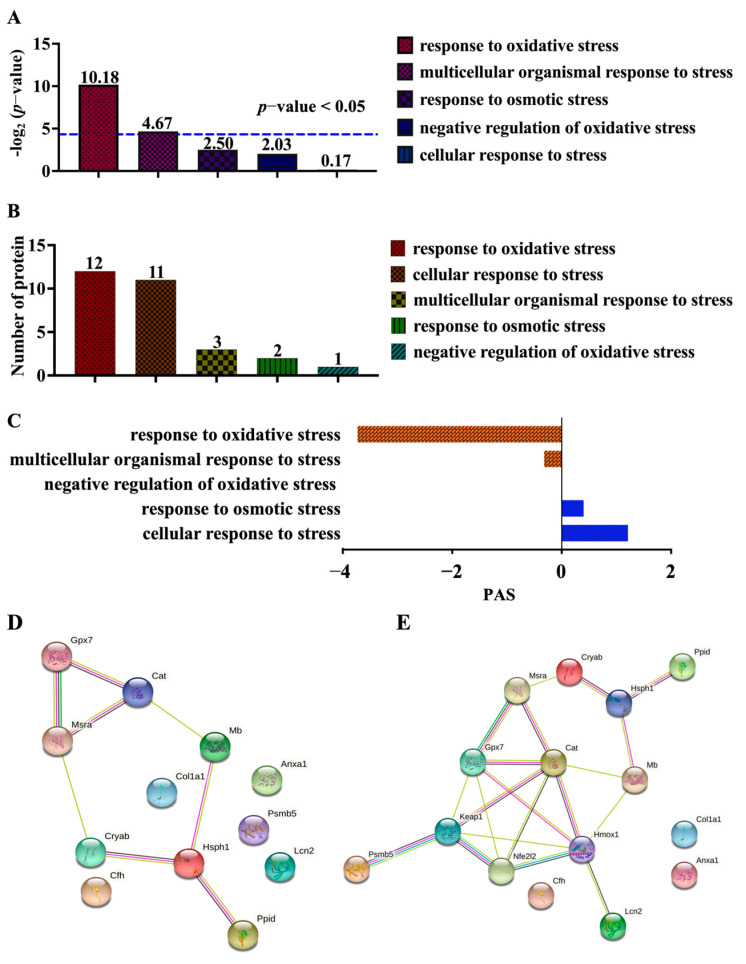
**The system of Nrf2 and antioxidant enzymes participated in WIBL.** (**A**) Analysis of significance of DEPs involved in response to stress. (**B**) Analysis of the number of DEPs involved in response to stress. (**C**) Analysis of effective intensity of DEPs involved in response to stress. (**D**) PPI network analysis of DEPs involved in response to oxidative stress. (**E**) PPI network analysis of DEPs that interact with the Nrf2 system. Nodes represent proteins, and edges represent predicted functional associations. Abbreviations: Nrf2, nuclear factor erythroid 2-related factor 2; WIBL: weightlessness-induced bone loss; PPI, protein–protein interaction; DEPs, differentially expressed proteins.

Stress response includes response to oxidative stress, multicellular organismal response to stress, response to osmotic stress, negative regulation of oxidative stress, and cellular response to stress. The significance of response to oxidative stress was the highest and the number of DEPs enriched in it were the largest (Figure 2A,B). In addition, the inhibitory effect of response to oxidative stress was the strongest (Figure 2C). Therefore, this study holds the opinion that response to oxidative stress exerted a vital role in WIBL. Protein–protein interaction (PPI) analysis of 12 DEPs in response to oxidative stress revealed that the closely related network of GPx7 (GSH-Px), CAT and Msra might be the main signaling pathway in regulating oxidative stress under SMG effect (Figure 2D). In order to clarify the main pathway involved in WIBL, other DEPs might be related to Gpx7, Cat and Msra were added on the basis of Figure 2D for further analysis, and a more complete pathway network was obtained (Figure 2E). Nrf2 (Nfe2l2), HO-1 (Hmox1) and Keap1 were found to be the main proteins interacting with Gpx7, Cat and Msra network. HO-1, GSH-Px and Cat are the downstream antioxidant enzymes regulated by the Keap1/Nrf2 signaling pathway. These data indicated that the Nrf2 system might change under the SMG effect and regulate the antioxidant enzymes system.

### 3.2. Effect of AMP1-1 Treatment on WIBL In Vivo

**Figure 3:**
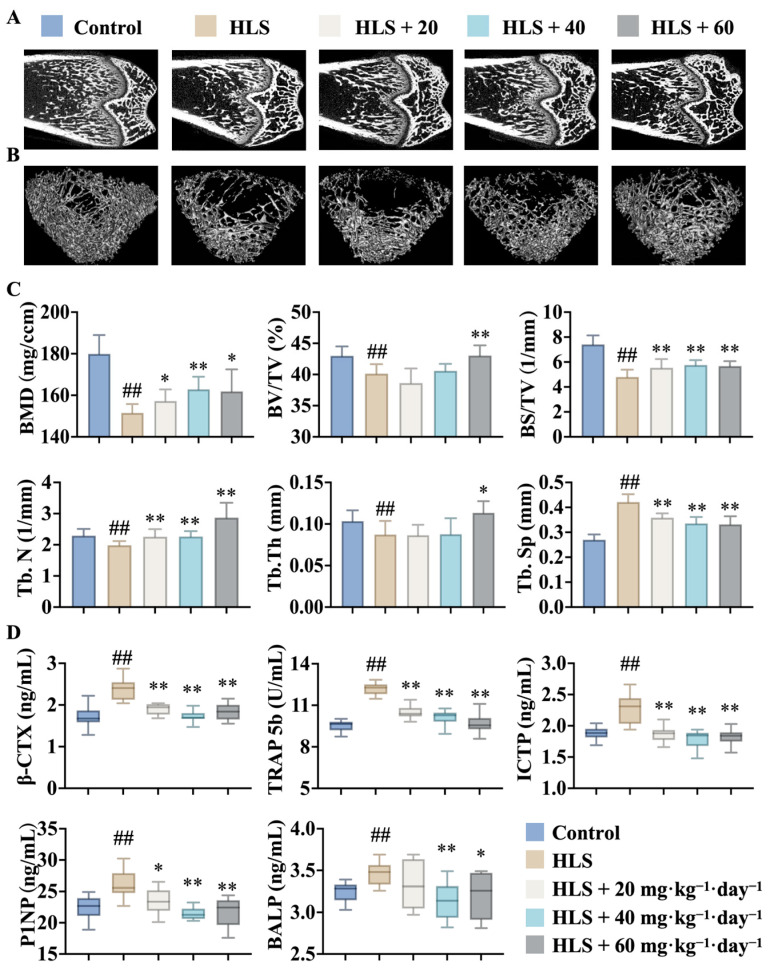
**Effect of AMP1-1 on femurs as evaluated by Micro CT and ELISA analysis.** (**A**) Representative three-dimensional reconstruction images of the distal femurs. (**B**) Representative images of trabecular bone microstructure. (**C**) Quantitative analysis of trabecular bone parameters, including BMD, BV/TV, BS/TV,Tb. N, Tb. Th and Tb. Sp. (**D**) Serum levels of bone turnover markers including β-CTX, TRAP 5b, ICTP, PINP and BALP measured by ELISA. Control: untreated normal rats; HLS: hindlimb-suspended rats; HLS + 20, HLS + 40, HLS + 60: HLS rats treated with AMP1-1 at 20, 40, or 60 mg·kg^−1^·day^−1^, respectively. Data are presented as mean ± SD (*n* = 6 per group). Statistical analysis was performed using one-way ANOVA followed by Tukey’s post hoc test. ## *p* < 0.01 vs. Control group; * *p* < 0.05, ** *p* < 0.01 vs. HLS group. Abbreviations: BMD: bone mineral density; BV/TV: bone volume fraction; BS/TV: bone surface density; Tb. Th, trabecular thickness; Tb. N, trabecular number; Tb. Sp, trabecular separation; β-CTX: cross-linked carboxyterminal telopeptide of type I collagen; TRAP 5b: tartrate-resistant acid phosphatase 5b; ICTP: cross-linked carboxyterminal telopeptide of type I collagen; P1NP: procollagen type 1 *N*-terminal propeptide; BALP: bone alkaline phosphatase.

Subsequently, we analyzed the protective effect of AMP1-1 on bone microstructure in rats. It could be inferred that AMP1-1 inhibited the bone loss caused by HLS (Figure 3A,B). Statistical analysis of cancellous bone related parameters showed that HLS could cause a decrease in Th. N, BV/TV, BS/TV and BMD and an increase in Tb. Sp, while AMP1-1 could reverse the changes (Figure 3C). In addition, we evaluated the protective effect of AMP1-1 on bone conversion factors such as β-CTX, TRAP 5b, ICTP, P1NP, and BALP. Statistically, it was found that the above factors in the serum of HLS rats were increased during the occurrence of bone loss. In contrast, AMP1-1 significantly improved the phenomenon (Figure 3D).

**Figure 4:**
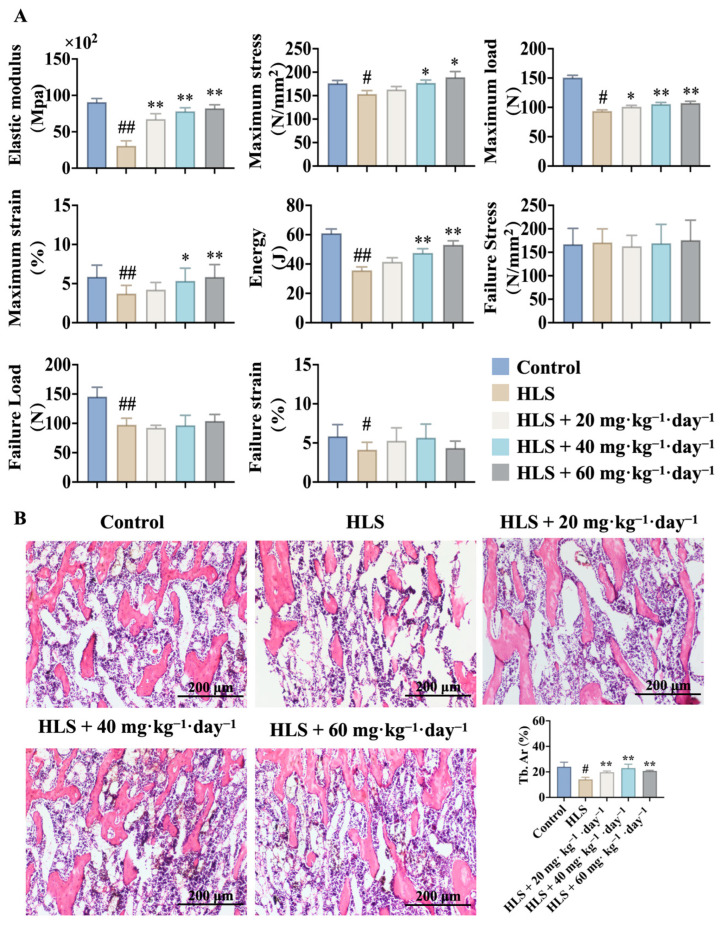
**Effect of AMP1-1 on femurs as evaluated by three-point bending and H&E staining.** (**A**) Biomechanical properties of femurs assessed by three-point bending test, including elastic modulus, maximum stress, maximum load, maximum strain, energy, failure stress, failure load and failure strain. (**B**) Representative images of H&E staining of femoral sections. Bars: 100 µm. Control: untreated normal rats; HLS: hindlimb-suspended rats; HLS + 20, HLS + 40, HLS + 60: HLS rats treated with AMP1-1 at 20, 40, or 60 mg·kg^−1^·day^−1^, respectively. Data are presented as mean ± SD (*n* = 6 per group). Statistical analysis was performed using one-way ANOVA followed by Tukey’s post hoc test. # *p* < 0.05, ## *p* < 0.01 vs. Control group; * *p* < 0.05, ** *p* < 0.01 vs. HLS group. Abbreviation: Tb. Ar, trabecular bone area percentage.

The femur plays a vital role in body support, movement and protection, and its biomechanical parameters are directly related to the state and function of the skeleton [25]. To prove the influence of AMP1-1 on bone mechanical properties, we analyzed and summarized the biomechanical parameters of femur. As shown in Figure 4A, elastic modulus, maximum stress, maximum load, maximum strain, energy, failure load and failure strain of HLS rats were significantly reduced compared to the control rats, while AMP1-1 could effectively improve them. The results of H&E staining demonstrated that there were large pyknosis of nuclei in the trabeculae and diffused empty lacunae in the medullary spaces in the HLS rats, accompanied by a significant reduction in Tb. Ar, while AMP1-1 treatment markedly improved these histopathological alterations (Figure 4B). These data suggested that AMP1-1 had a significant protective effect on WIBL in vivo.

### 3.3. AMP1-1 Maintained Bone Homeostasis by Inhibiting Oxidative Stress In Vivo

**Figure 5:**
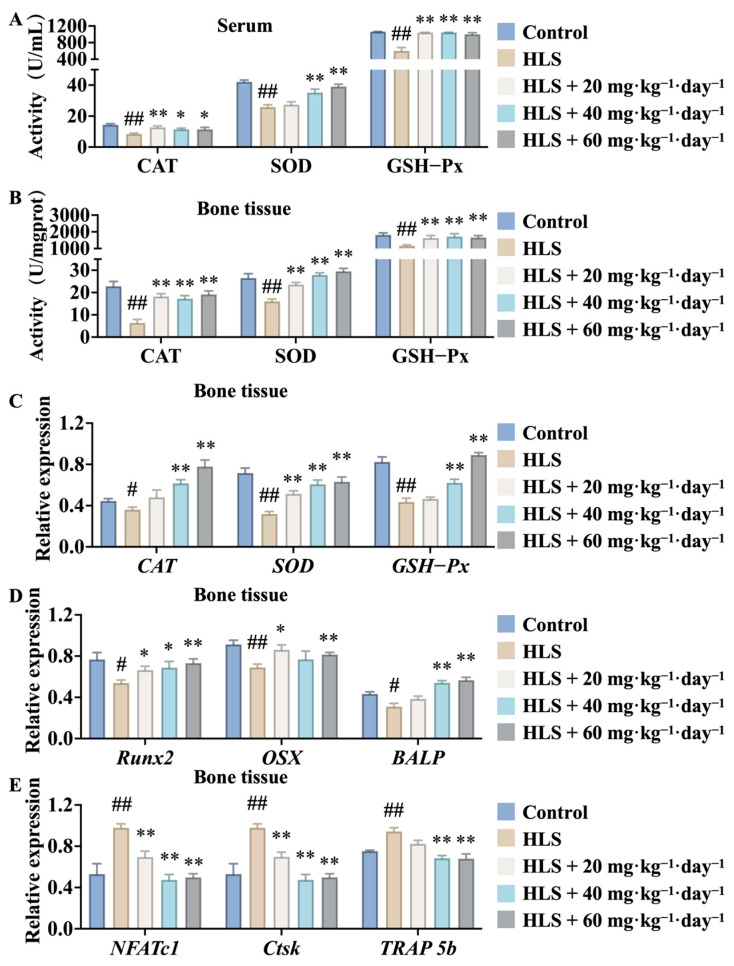
**AMP1-1 maintained bone homeostasis by inhibiting oxidative stress in vivo.** (**A**) Activities of antioxidant enzymes SOD, CAT, and GSH-Px in serum. (**B**) Activities of SOD, CAT, and GSH-Px in femoral tissues. (**C**) Relative mRNA expression of *SOD*, *CAT*, and *GSH-Px* in bone tissue, normalized to *GAPDH*. (**D**) Relative mRNA expression of osteoblastic marker genes *Runx2*, *OSX* and *BALP* in bone tissue. (**E**) Relative mRNA expression of osteoclastic marker genes *NFATc1*, *Ctsk* and TRAP 5b in bone tissue. Control: untreated normal rats; HLS: hindlimb-suspended rats; HLS + 20, HLS + 40, HLS + 60: HLS rats treated with AMP1-1 at 20, 40, or 60 mg·kg^−1^·day^−1^, respectively. Data are presented as mean ± SD (*n* = 6 per group). Statistical analysis was performed using one-way ANOVA followed by Tukey’s post hoc test. # *p* < 0.05, ## *p* < 0.01 vs. Control group; * *p* < 0.05, ** *p* < 0.01 vs. HLS group. Abbreviations: SOD, superoxide dismutase; CAT, catalase; GSH-Px, glutathione peroxidase; Runx2, runt-related transcription factor 2; OSX, osterix; BALP, bone alkaline phosphatase; NFATc1, nuclear factor of activated T-cells c1; Ctsk, cathepsin K; TRAP 5b, tartrate-resistant acid phosphatase 5b.

To confirm the results in omics, the CAT, SOD and GSH-Px activities in serum and femurs were detected. It was found that the enzymes in HLS rats were decreased while the down-regulation was inhibited by AMP1-1 (Figure 5A,B). Compared to the control group, the gene expression of CAT, SOD and GSH-Px in HLS rats were down-regulated. However, they were recovered after the treatment of AMP1-1 (Figure 5C). Combined with the above results, we concluded that HLS caused bone loss and AMP1-1 inhibited the occurrence of bone loss by promoting the activity of antioxidant enzymes. In addition, the gene expression of Runt-related transcription factor 2 (*Runx2*), Osterix (*OSX*) and *BALP* were down-regulated in HLS rats, but increased in the AMP1-1 treated rats (Figure 5D). The gene expression of nuclear factor of activated T-cells c1 (*NFATc1*), cathepsin K (*Ctsk*) and TRAP 5b were significantly increased in HLS rats, but significantly inhibited by AMP1-1 (Figure 5E).

### 3.4. AMP1-1 Protected Against WIBL by Regulating the Nrf2/HO-1 Pathway in Rats

**Figure 6:**
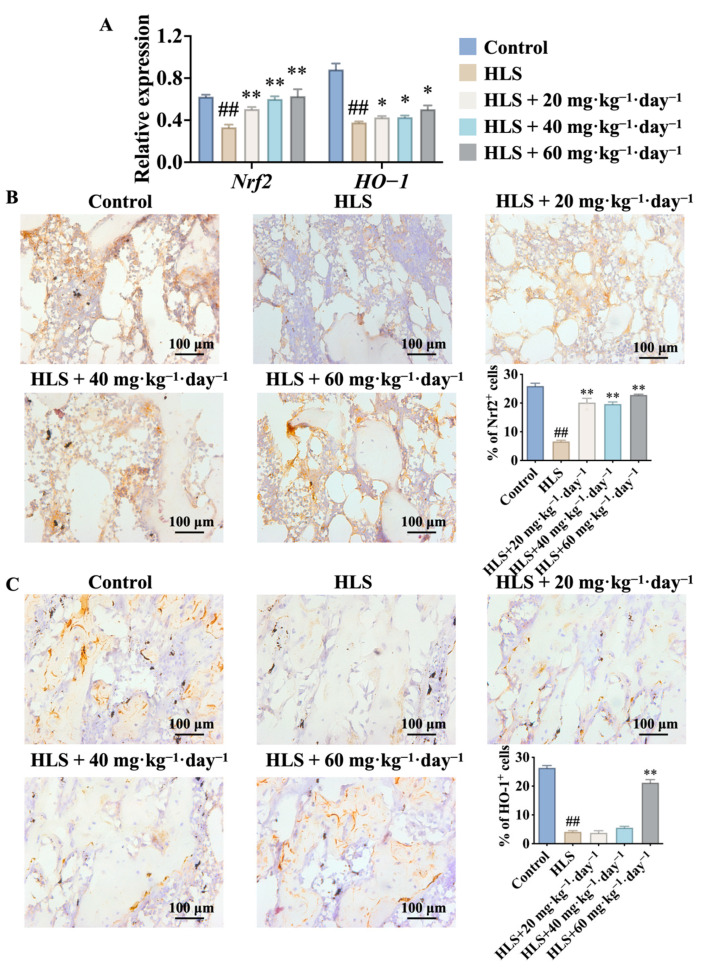
**AMP1-1 protected against WIBL by regulating the Nrf2/HO-1 pathway in rats.** (**A**) Relative mRNA expression of *Nrf2* and *HO-1* in bone tissue, normalized to *GAPDH*. (**B**) Representative immunohistochemical images and quantitative analysis of Nrf2-positive cells in bone tissue. (**C**) Representative immunohistochemical images and quantitative analysis of HO-1-positive cells in bone tissue. Control: untreated normal rats; HLS: hindlimb-suspended rats; HLS + 20, HLS + 40, HLS + 60: HLS rats treated with AMP1-1 at 20, 40, or 60 mg·kg^−1^·day^−1^, respectively. Data are presented as mean ± SD (*n* = 6 per group). Statistical analysis was performed using one-way ANOVA followed by Tukey’s post hoc test. ## *p* < 0.01 vs. Control group; * *p* < 0.05, ** *p* < 0.01 vs. HLS group. Abbreviations: Nrf2, nuclear factor erythroid 2-related factor 2; HO-1, heme oxygenase-1.

It was indicated that the gene expression of *Nrf2* and *HO-1* was decreased in HLS rats, but significantly improved after AMP1-1 treatment (Figure 6A). Consist with the above results, it was found that the positive cells of Nrf2 or HO-1 were decreased in bone tissue of HLS rats. The treatment with AMP1-1 could improve the phenomenon (Figure 6B,C). These results verified the above hypothesis that AMP1-1 could mediate the Nrf2 pathway to regulate antioxidant enzyme activity and protect against WIBL.

### 3.5. AMP1-1 Alleviated the Bone Homeostasis Imbalance Caused by SMG In Vitro

**Figure 7:**
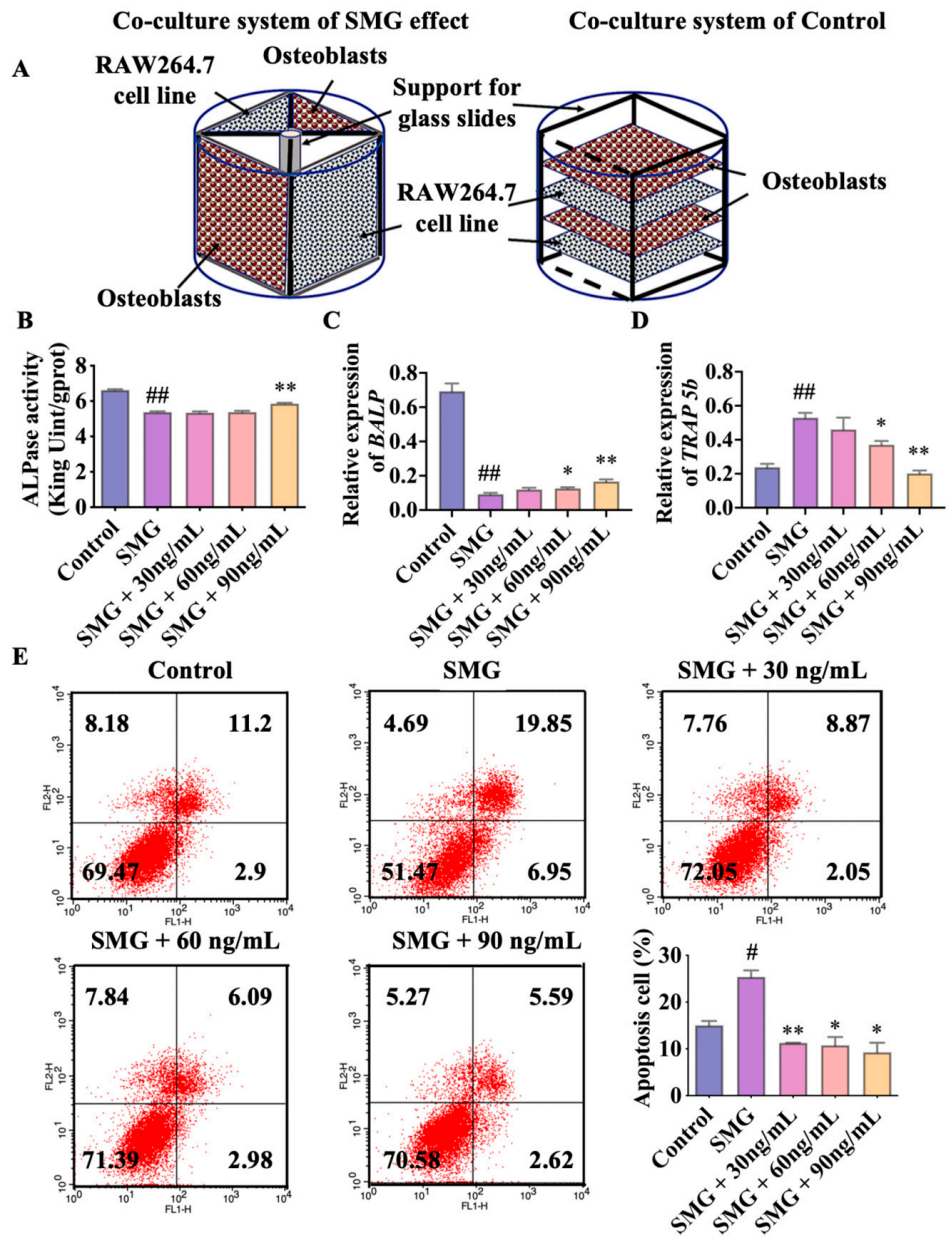
**AMP1-1 alleviated the bone homeostasis imbalance caused by SMG in vitro.** (**A**) Schematic diagram of the experimental design and SMG treatment in the co-culture system. (**B**) ALP activity in osteoblasts. (**C**) Relative mRNA expression of *BALP* in osteoblasts, normalized to *GAPDH*. (**D**) Relative mRNA expression of TRAP 5b in RAW264.7 cell line, normalized to *GAPDH*. (**E**) Representative images and quantitative analysis of osteoblast apoptosis. Control: cells cultured under normal gravity; SMG: cells exposed to simulated microgravity; SMG + 30, SMG + 60, SMG + 90: SMG-exposed cells treated with AMP1-1 at 30, 60, or 90 ng/mL, respectively. Data are presented as mean ± SD (*n* = 3 per group). Statistical analysis was performed using one-way ANOVA followed by Tukey’s post hoc test. # *p* < 0.05, ## *p* < 0.01 vs. Control group; * *p* < 0.05, ** *p* < 0.01 vs. SMG group. Abbreviations: SMG, simulated microgravity; ALP, alkaline phosphatase; BALP, bone alkaline phosphatase; TRAP 5b, tartrate-resistant acid phosphatase 5b.

In the co-culture system, RAW264.7 cell line might be stimulated into osteoclasts induced by osteoblasts-secreted cytokines [11]. The experimental design and the treatment of SMG are demonstrated in Figure 7A. It was exhibited that the enzyme activity and the gene expression of BALP in osteoblasts were decreased under the SMG conditions, while AMP1-1 had positive effect on them (Figure 7B,C). The gene expression of TRAP 5b in osteoclasts was significantly increased under the SMG effect while AMP1-1 inhibited it (Figure 7D). In addition, AMP1-1 also alleviated the apoptosis induced by weightlessness in osteoclasts (Figure 7E).

### 3.6. AMP1-1 Exerted a Protective Role in Cell Co-Culture System by Inhibiting Oxidative Stress

**Figure 8:**
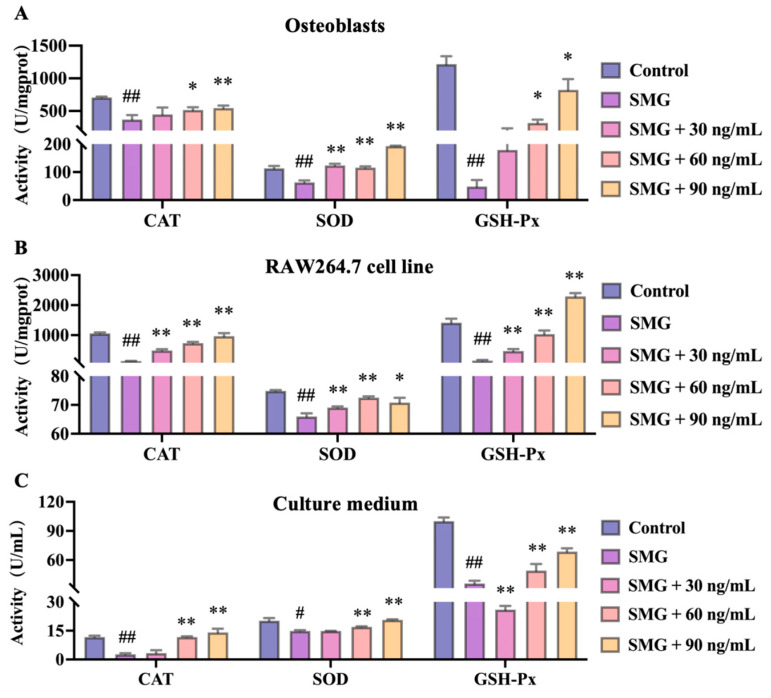
**AMP1-1 exerted a protective influence in the cell co-culture system by inhibiting oxidative stress.** (**A**) Activities of SOD, CAT, and GSH-Px in osteoblasts. (**B**) Activities of SOD, CAT, and GSH-Px in RAW264.7 cell line. (**C**) Activities of SOD, CAT, and GSH-Px in the culture medium. Control: cells cultured under normal gravity; SMG: cells exposed to simulated microgravity; SMG + 30, SMG + 60, SMG + 90: SMG-exposed cells treated with AMP1-1 at 30, 60, or 90 ng/mL, respectively. Data are presented as mean ± SD (*n* = 3 per group). Statistical analysis was performed using one-way ANOVA followed by Tukey’s post hoc test. # *p* < 0.05, ## *p* < 0.01 vs. Control group; * *p* < 0.05, ** *p* < 0.01 vs. SMG group. Abbreviations: SMG, simulated microgravity; SOD, superoxide dismutase; CAT, catalase; GSH-Px, glutathione peroxidase.

To further understand the protective mechanism of AMP1-1, the antioxidant enzymes in the co-culture system were investigated. It worth noting that the CAT, SOD and GSH-Px activities in osteoblasts were diminished under the SMG effect while AMP1-1 presented strong antioxidant activity in osteoblasts (Figure 8A). Interestingly, the antioxidant enzymes of osteoclasts and culture medium were also decreased significantly, while AMP1-1 increased the antioxidant enzyme activity (Figure 8B,C). The results indicated that AMP1-1 could mitigate the weightless effect by increasing the activities of antioxidant enzymes.

### 3.7. AMP1-1 Protected Against WIBL by Regulating the Nrf2/HO-1 Pathway in the Co-Culture System

**Figure 9:**
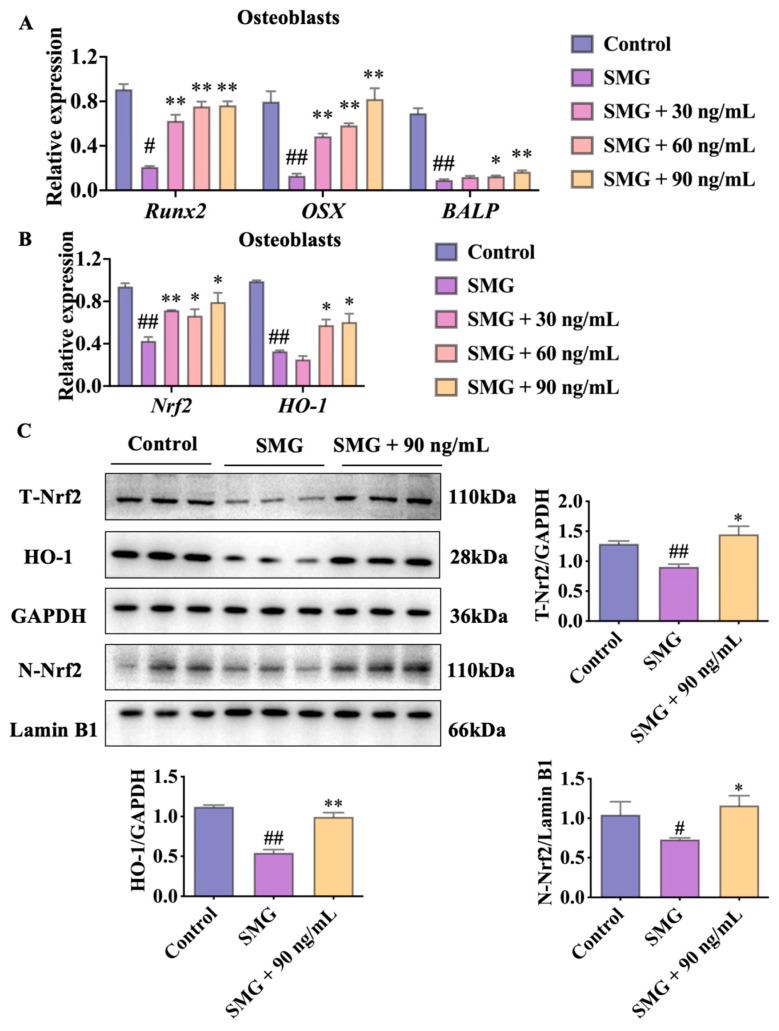
**AMP1-1 protected against WIBL by regulating the Nrf2/HO-1 pathway in osteoblasts.** (**A**) Relative mRNA expression of osteoblastic marker genes *Runx2*, *OSX* and *BALP* in osteoblasts, normalized to *GAPDH*. (**B**) Relative mRNA expression of *Nrf2* and *HO-1* in osteoblasts, normalized to *GAPDH*. (**C**) Protein expression levels of Nrf2 and HO-1 in osteoblasts determined by Western blot, with GAPDH or Lamin B1 as the loading control. Control: cells cultured under normal gravity; SMG: cells exposed to simulated microgravity; SMG + 30, SMG + 60, SMG + 90: SMG-exposed cells treated with AMP1-1 at 30, 60, or 90 ng/mL, respectively. Data are presented as mean ± SD (*n* = 3 per group). Statistical analysis was performed using one-way ANOVA followed by Tukey’s post hoc test. # *p* < 0.05, ## *p* < 0.01 vs. Control group; * *p* < 0.05, ** *p* < 0.01 vs. SMG group. Abbreviations: SMG, simulated microgravity; Nrf2, nuclear factor erythroid 2-related factor 2; HO-1, heme oxygenase-1; Runx2, runt-related transcription factor 2; OSX, osterix; BALP, bone alkaline phosphatase.

**Figure 10:**
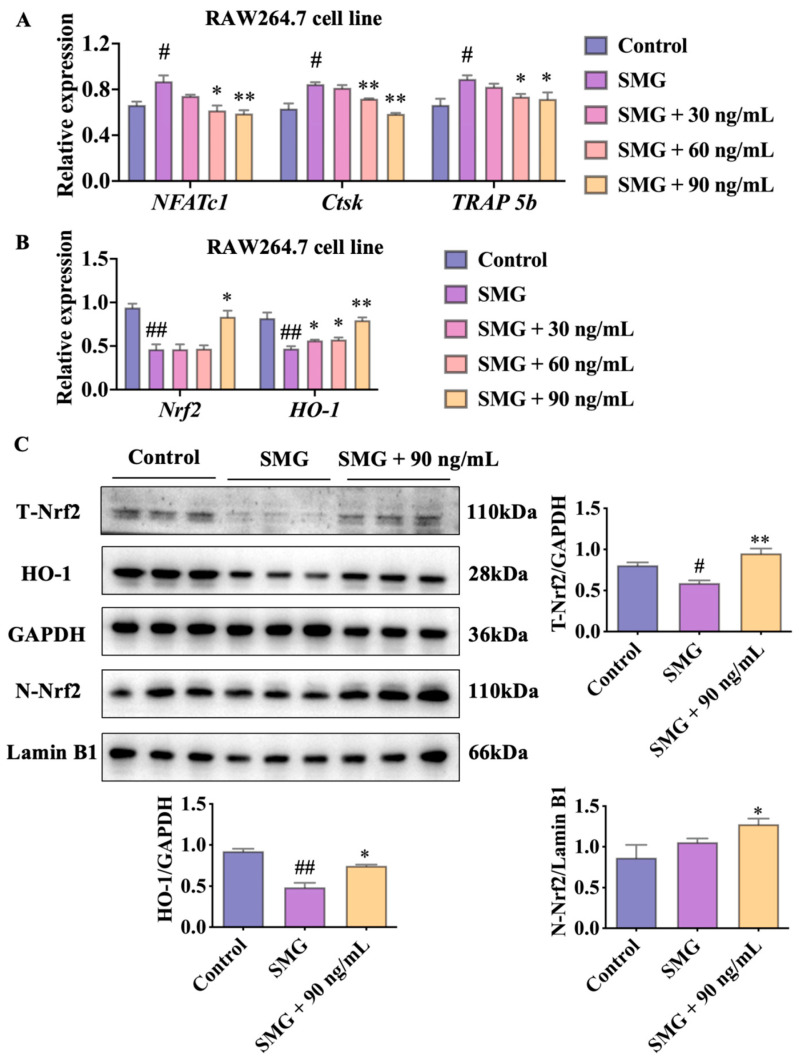
**AMP1-1 protected against WIBL by regulating the Nrf2/HO-1 pathway in RAW264.7 cell line.** (**A**) Relative mRNA expression of osteoclastic marker genes *NFATc1*, *Ctsk* and TRAP 5b in RAW264.7 cell line, normalized to *GAPDH*. (**B**) Relative mRNA expression of *Nrf2* and *HO-1* in RAW264.7 cell line, normalized to *GAPDH*. (**C**) Protein expression levels of Nrf2 and HO-1 in RAW264.7 cell line determined by Western blot, with GAPDH or Lamin B1 as the loading control. Control: cells cultured under normal gravity; SMG: cells exposed to simulated microgravity; SMG + 30, SMG + 60, SMG + 90: SMG-exposed cells treated with AMP1-1 at 30, 60, or 90 ng/mL, respectively. Data are presented as mean ± SD (*n* = 3 per group). Statistical analysis was performed using one-way ANOVA followed by Tukey’s post hoc test. # *p* < 0.05, ## *p* < 0.01 vs. Control group; * *p* < 0.05, ** *p* < 0.01 vs. SMG group. Abbreviations: SMG, simulated microgravity; Nrf2, nuclear factor erythroid 2-related factor 2; HO-1, heme oxygenase-1; NFATc1, nuclear factor of activated T-cells c1; Ctsk, cathepsin K; TRAP 5b, tartrate-resistant acid phosphatase 5b.

Based on the potential of AMP1-1 in anti-WIBL, its activity was verified and its protective pathway was explored at the cellular level. It was shown that under SMG conditions, the gene expression of *Runx2*, *OSX* and *BALP* in osteoblasts were down-regulated, while AMP1-1 reversed the changes (Figure 9A). In addition, the genes regulation of *Nrf2* and *HO-1* in SMG osteoblasts were significantly inhibited, while AMP1-1 improved the above phenomenon (Figure 9B). In osteoblasts, the total Nrf2, HO-1 and the Nrf2 protein in the nucleus decreased significantly under the SMG effect, while 90 ng/mL AMP1-1 improved the above phenomenon (Figure 9C). Finally, this study determined the changes in RAW264.7 cell line and found that the gene expression of *NFATc1*, *Ctsk* and TRAP 5b in SMG cells were significantly increased, while AMP1-1 inhibited the expression (Figure 10A). The alteration of Nrf2 and HO-1 levels in RAW264.7 cell line are similar to those in osteoblasts. Under the SMG conditions, the total Nrf2, HO-1 and Nrf2 protein in the nucleus decreased significantly while 90 ng/mL AMP1-1 improved them (Figure 10B,C). In conclusion, AMP1-1 plays antioxidant effect through the Nrf2/HO-1 regulation in osteoblasts and RAW264.7 cell line, thereby providing protection against WIBL.

## 4. Discussion

Bone loss occurs in astronaut during flight missions, and it is difficult to restore to their pre-flight state [26]. Therefore, it is urgent to probe the pathological mechanism of WIBL and explore the new treatment strategies [27]. ITRAQ technology has gradually used to explore the mechanisms of disease development [28]. Peng [29] found that ASPH had positive correlations with osteogenic markers and regulate Wnt signaling mediated by Gsk3 beta. In addition, OZCAN [30] took advantage of iTRAQ to determine the pathways of senescence-associated secretory phenotype. In this study, iTRAQ and PPI analysis revealed that the network of Nrf2 system interact with CAT, SOD, GSH-Px played an important role in WIBL. It has shown that Loureirin B disturbs osteoclast genesis and osteoporosis by suppressing ROS levels [31]. In addition, it has found that Pseurotin A inhibits bone loss by suppressing oxidative stress [32]. ROS plays a crucial role in bone formation and bone resorption while CAT is one of the key enzymes in scavenging ROS [33]. Therefore, our study focused on the network of Nrf2 system interact with CAT, SOD, GSH-Px in the subsequent experiments.

In our previous study, the pro-proliferation effect of polysaccharide extracted from seven natural products on osteoblasts were investigated. AMP was proved to have the strongest promotional effect on cell proliferation [34]. Subsequently, the active fragment AMP1-1 was purified from AMP and its protective mechanism on WIBL were explored in this study. The cancellous bone can be conducted by 3D imaging, BMD, BV/TV, BS/TV, Tb. N, Tb. Th and Tb. Sp [35]. BMD represents the mineral quality per unit of bone mass in bone tissue and is a direct indicator for clinical evaluation of bone loss [36]. BV/TV reflects the bone mass and BS/TV is the ratio of surface area to volume of trabecular bone, which can reflect the condition of bone remodeling [37]. In addition, Tb. N, Tb. Th and Tb. Sp are used to evaluate the spatial morphology and structure of trabecular bone [38]. In this study, BMD, BV/TV, BS/TV, Tb. N, Tb. Th were decreased and Tb. Sp was increased significantly in HLS rats, while AMP1-1 reversed the changes. The femur exerts a role in maintaining the skeletal function due to its biomechanical properties. The commonly used indicators of bone biomechanic include failure stress, failure load, failure stress, elastic modulus, maximum stress, maximum load, maximum strain and energy [39]. It was indicated that the above indexes in HLS rats were reduced, while they were effectively improved by AMP1-1. Moreover, the H&E staining results indicated that AMP1-1 alleviated pathological damage in bone tissue.

Bone homeostasis is maintained through the coordinated actions of osteoblasts and osteoclasts [40]. TRAP 5b, β-CTX and ICTP are bone turnover markers for evaluating bone resorption [41]. These markers were increased in the serum of HLS rats, indicating that HLS can promote bone resorption and lead to bone loss. However, AMP1-1 treatment effectively alleviated the changes. Moreover, ICTP and P1NP can directly reflect bone turnover and the content of P1NP in serum is elevated in bone metabolism disease [42]. BALP is a marker to assess osteogenic activity, and it is increased in bone metabolism disease [43]. In the present study, ICTP, BALP and P1NP were extremely increased in HLS rats, indicating that HLS could significantly increase the bone turnover rate in rats. SMG-induced simultaneous elevation of bone turnover markers reflects a high-turnover state characterized by uncoupling between bone resorption and bone formation in serum. Although bone formation is increased, it is far from sufficient to compensate for the enhanced bone resorption, ultimately leading to net bone loss. This interpretation is consistent with previous findings in both spaceflight and ground-based microgravity models where skeletal unloading leads to uncoupling of bone formation and resorption, with resorption predominating over formation [2]. In addition, AMP1-1 was found to exert an inhibitory role in ICTP, P1NP and BALP contents.

The previous study showed that polygonum polysaccharide exerted a preventive effect on bone loss by improving BMD and bone microstructure destruction in ovariectomized rats [44]. In addition, it was found that Achyranthes bidentata polysaccharide could treat bone loss by modulating bone metabolic factors and improving bone microarchitecture [45]. Compared with the mentioned polysaccharides, a low concentration of AMP1-1 at 60 mg kg^−1^ day^−1^ in our study could attenuate WIBL as assessed by the bone microstructure, biomechanical properties and bone turnover indexes. Oxidative stress is a key driver of the imbalance between bone formation and resorption [46]. The detection of antioxidant enzymes and gene expression in rats showed that AMP1-1 increased the CAT, SOD and GSH-Px levels. Given that the above enzymes are regulated by Keap1/Nrf2 signaling pathway, and Nrf2 is the key transcription factor participating in oxidative stress [47], the levels of Nrf2 and HO-1 were examined. The results indicated that Nrf2 and HO-1 were declined in HLS rats, while AMP1-1 could increase them. At the same time, AMP1-1 promoted the expression of osteogenic marker genes and suppressed the expression of osteoclastic marker genes. These findings are consistent with the known role of AMPK in regulating oxidative stress during osteoclast differentiation [48]. This suggests that AMP1-1 may restore bone metabolic balance by enhancing the antioxidant capacity of bone tissue.

AMP1-1 can be directly absorbed into the blood through intestinal mucosa as a kind of inulin polysaccharide [49]. In the current study, osteoblasts and RAW264.7 cell line were co-cultured in a 2D-RWVS bioreactor simulating microgravity, allowing the cells to interact in a shared environment to exhibit high concordance with in vivo conditions [50]. The results in cell co-culture system showed AMP1-1 could promote osteogenic differentiation, reduce cell apoptosis in osteoblasts and inhibit osteoclastic differentiation. Previous studies have indicated that 50 μg/mL Achyranthes bidentata polysaccharides could promote proliferation and ALP activity of MC3T3-E1 cells [51]. In addition, it was found that the pretreatment of MC3T3-E1 cells with 800 µg/mL Lycium barbarum polysaccharides could effectively inhibit apoptosis in osteoblasts [52]. Compared with the above polysaccharides, the low dose of 30 ng/mL AMP1-1 could have a significant regulatory effect on co-culture model. Our data collectively demonstrate that AMP1-1 exerts a dual regulatory effect on bone remodeling. AMP1-1 promotes osteogenic activity and enhances bone formation. This is supported by our observations of increased bone formation markers and enhanced osteogenic gene expression in both bone tissues and osteoblasts. AMP1-1 inhibits osteoclastic activity and suppresses bone resorption. This is evidenced by the decreased resorption markers and reduced osteoclast-related gene expression in both bone tissues and RAW264.7 cell line. Thus, AMP1-1 shifts the bone remodeling balance toward bone formation by simultaneously promoting bone formation and inhibiting bone resorption, thereby re-establishing the equilibrium disrupted by SMG. This dual mechanism is particularly significant in the context of simulated microgravity, where the normal coupling between formation and resorption is disrupted. By restoring the balance, AMP1-1 effectively counteracts the uncoupling induced by mechanical unloading.

AMP1-1 is an inulin-type fructan containing glucose and fructose; it can produce a free aldehyde to reduce free radicals, thus exerting strong antioxidant activity [11]. The antioxidant enzymes were decreased in osteoblasts, RAW264.7 cell line and cell culture medium under simulating microgravity, while AMP1-1 increased them. In addition, it was found that inulin polysaccharides could activate the Keap1/Nrf2 signaling pathway [53]. The content of Nrf2 and HO-1 in osteoblasts and RAW264.7 cell line were decreased, while AMP1-1 increased the nuclear translocation of Nrf2 in the above cells and enhanced the levels of HO-1 protein. Therefore, we hypothesized that AMP1-1 might scavenge excessive free radicals and resist oxidative stress in vivo by mediating Nrf2 pathway and catalyzing the transcription of HO-1. It was reported that the increased HO-1 promoted the expression of Runx2 [54]. OSX is an important transcription factor regulating osteogenic differentiation, while Runx2 can directly mediate the transcription of OSX [55]. Moreover, ALP is a downstream enzyme of Runx2 and Osx to regulate the activity of osteoblasts [56]. Our results investigated that AMP1-1 promoted Runx2, Osx and ALP expression through Nrf2 pathway. As an important transcription factor, NFATc1 regulates the gene expression of osteoclastic differentiation including Ctsk and TRAP 5b [57]. In this study, AMP1-1 inhibited the expression of NFATc1, Ctsk and TRAP 5b, which also demonstrated that AMP1-1 reduced bone resorption via Nrf2 signaling pathway.

It was shown that 5 μM Cistanche deserticola polysaccharide attenuated bone resorption by reducing ROS production [58]. In addition, 5 μM Poria cocos polysaccharide [59] and 6.5 μM Achyranthes bidentata polysaccharide [60] can suppress osteoclastic differentiation by inhibiting RANKL signaling. Compared with the above-mentioned research, 90 ng/mL (0.06 μM) AMP1-1 could significantly regulate osteoblasts and RAW264.7 cell line. Compared with the other polysaccharides, the low dose of 90 ng/mL AMP1-1 with such efficacy might be due to its low molecular weight. Our previous research found that the relative molecular mass of AMP1-1 was 1433 [11]. It has shown that the fucoidan with low molecular weight exhibits stronger effect on bone formation [61]. Hence, we hold the opinion that the molecular weight of AMP1-1 exerted an vital role in WIBL and the polysaccharides with low molecular weight had more potential to resist WIBL. AMP1-1 could directly enter the blood circulation to act on osteoblasts and osteoclasts in vitro. It might alleviate WIBL by regulating the Nrf2 pathway and defensing oxidative stress. Nevertheless, this study also has some limitations. The present findings mainly demonstrate an association between AMP1-1 treatment and regulation of the Nrf2/HO-1 pathway. In view of that Nrf2 knockdown or pharmacological inhibition was not performed, the causal contribution of Nrf2 to the protective effects of AMP1-1 remains to be established. Future studies employing these mechanistic approaches are warranted to determine whether Nrf2 activation is necessary for AMP1-1-mediated protection against WIBL. These plans include utilizing specific Nrf2 inhibitors or Nrf2-siRNA knockdown approaches, combined with rescue experiments, to further interrogate the causal role of this pathway. The specific receptor for AMP1-1 has not yet been definitively identified in our current study. AMP1-1 is a novel polysaccharide isolated and purified in our previous study, and its detailed molecular targeting remains an ongoing focus of our research. Nevertheless, based on the regarding polysaccharide bioactivity, we hypothesize that AMP1-1 may act through Toll-like receptors (TLRs) such as TLR4 or TLR2, as these are the primary pattern recognition receptors for most natural polysaccharides [62]. Importantly, this hypothesis is indirectly supported by our own data. In RAW264.7 cell line, AMP1-1 mediates the Nrf2 signaling pathway, which is known to intersect with TLR4-mediated inflammatory and oxidative stress responses [63]. The activation of downstream antioxidant enzymes following AMP1-1 treatment is consistent with the reported downstream effects of TLR4 engagement by other plant-derived polysaccharides [64]. The beneficial effects of AMP1-1 on astronauts must be supported by human-relevant evidence. Currently, we can only state that AMP1-1 shows promising efficacy in well-established rodent models of SMG, which have been widely used as preclinical surrogates for spaceflight-induced bone loss. In the subsequent experiments, we plan to evaluate the effect of AMP1-1 on human peripheral blood mononuclear cells or human osteoblast-like cell lines to assess its cross-species activity. TLR4/TLR2 gene-knockout or humanized TLR-transgenic mouse models will be utilized to definitively identify the receptor responsible for AMP1-1. It should be noted that the present study evaluated the effect of AMP1-1 on osteoclasts primarily in the co-culture system. While this approach better reflects the physiological bone microenvironment, whether AMP1-1 exerts a direct effect on osteoclasts in monoculture remains to be determined. Future studies will investigate the direct effect of AMP1-1 on osteoclast differentiation and activity in monoculture to further dissect its cell-type-specific mechanisms. It should be noted that the present study did not systematically measure bone wet weight or muscle weights that are well-recognized as critical indices in antigravity research. In addition, although our organ coefficient data (Supplementary Figure S3B) preliminarily suggest that AMP1-1 has no obvious toxicity on major organs, and natural polysaccharides are generally considered to have low toxicity, we did not perform systematic toxicological evaluation of AMP1-1 in the current study. Future studies will comprehensively assess these physiological parameters and conduct thorough safety evaluations, including acute and sub-chronic toxicity tests, to establish the safety and efficacy profile of AMP1-1 for potential translational applications. Another limitation of the present study is the absence of a positive control in both the in vivo and in vitro experiments. The primary aim of this study was to conduct an initial evaluation of the pharmacological effects of AMP1-1 and to determine whether it could alleviate HLS-induced bone loss. Therefore, the experimental design mainly focused on comparing the HLS group with different doses of AMP1-1. However, without a positive control, the efficacy of AMP1-1 cannot be directly compared with that of established therapeutic interventions. Future studies should include appropriate positive controls and further investigate the specific molecular targets and mechanisms of AMP1-1.

## 5. Conclusions

In this study, we first identified that DEPs in HLS rats were enriched in the oxidative stress and Nrf2/HO-1 pathways. AMP1-1 treatment improved bone microstructure, biomechanical properties, and inhibited bone turnover markers in HLS rats. It also enhanced antioxidant enzyme activities, promoted osteogenic genes, and suppressed osteoclast genes. In a co-culture model, AMP1-1 promoted osteoblastic differentiation, inhibited osteoclastic differentiation, and activated the Nrf2/HO-1 pathway. Thus, AMP1-1 alleviates WIBL by activating the Nrf2/HO-1 pathway and reducing oxidative stress. The molecular mechanism by which AMP1-1 exerted a protective role in WIBL was revealed in this study and it has theoretical and practical significance for the further development of anti-WIBL agents.

## Data Availability

Data will be made available on request.

## Supplementary Materials

The following supporting information can be downloaded at https://www.mdpi.com/article/10.3390/nu18152566/s1, Figure S1: A flow chart of iTRAQ analysis to show the procedure of sample preparation, liquid chromatography coupled with LC-MS/MS analysis, database search and bioinformatics analysis; Figure S2: Statistical analysis of Micro CT parameters; Figure S3: Effect of AMP1-1 on body weight growth and organ index; Table S1: The information of differential expressed proteins in response to oxidative stress; Table S2: Effects of AMP1-1 on bone biomechanical parameters in femurs as assessed by three-point bending; Table S3: Primer sequences used for qRT-PCR analysis.

## Abbreviations

WIBL	Weightlessness-induced bone loss
AMP	Atractylodes macrocephala polysaccharide
HLS	Hindlimb suspension
Nrf2	Nuclear factor erythroid 2-related factor 2
HO-1	Heme oxygenase-1
ROS	Reactive oxygen species
Keap1	Kelch-like ECH-associated protein 1
SOD	Superoxide dismutase
SD	Sprague-Dawley
Micro CT	Microcomputed Tomography
ITRAQ	Isobaric tags for relative and absolute quantification
3D	Three-dimensional
BMD	Bone mineral density
BV/TV	Bone volume fraction
BS/TV	Bone surface density
Tb. N	Trabecular number
Tb. Th	Trabecular thickness
Tb. Sp	Trabecular separation
Tb. Ar	Trabecular bone area
DEPs	Differential expressed proteins
β-CTX	Cross-linked carboxyterminal telopeptide of type I collagen
TRAP 5b	Tartrate-resistant acid phosphatase 5b
ICTP	Cross-linked carboxyterminal telopeptide of type I collagen
P1NP	Procollagen type 1 *N*-terminal propeptide
BALP	Bone alkaline phosphatase
CAT	Catalase
GSH-Px	Glutathione peroxidase
ALP	Alkaline phosphatase
QRT-PCR	Quantificational real-time polymerase chain reaction
IHC	Immunohistochemical
SMG	Simulated microgravity
2D-RWVS	2D-Rotating Wall Vessel
GO	Gene ontology
KEGG	Kyoto encyclopedia of genes and genomes
BP	Biological process
PPI	Protein–protein interaction
Runx2	Runt-related transcription factor 2
OSX	Osterix
NFATc1	Nuclear factor of activated T-cells c1
Ctsk	Cathepsin K
