# Supplementary material for: An Inulin-Type Atractylodes Macrocephala Polysaccharide Alleviates Weightlessness-Induced Bone Loss in Association with the Nrf2/HO-1 Pathway
Source: Nutrients. 2026 Aug 6;18(15):2566. doi: 10.3390/nu18152566 (PMC13468469; doi:10.3390/nu18152566)
Supplement: Supplementary file 1 [file nutrients-18-02566-s001.zip › nutrients-4423398-supplementary.pdf]

Supplementary File

1.1 An overview of isobaric tags for relative and absolute quantification (iTRAQ) analysis

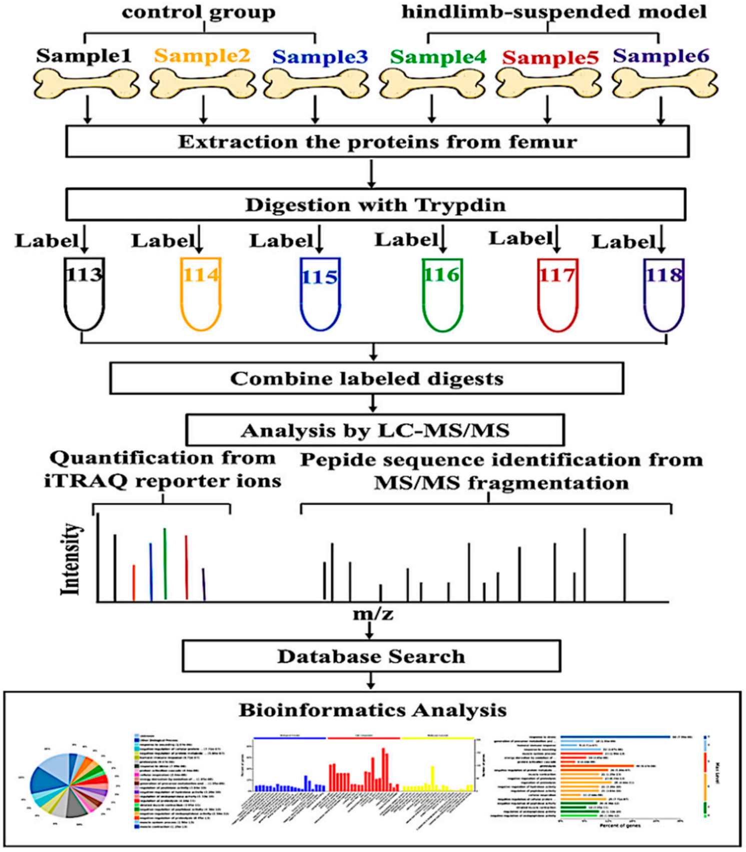

Figure S1. A flow chart of iTRAQ analysis to show the procedure of sample preparation, liquid chromatography coupled with LC-MS/MS analysis, database search and bioinformatics analysis.

1.2 Statistical analysis of Micro CT in Figure 1A

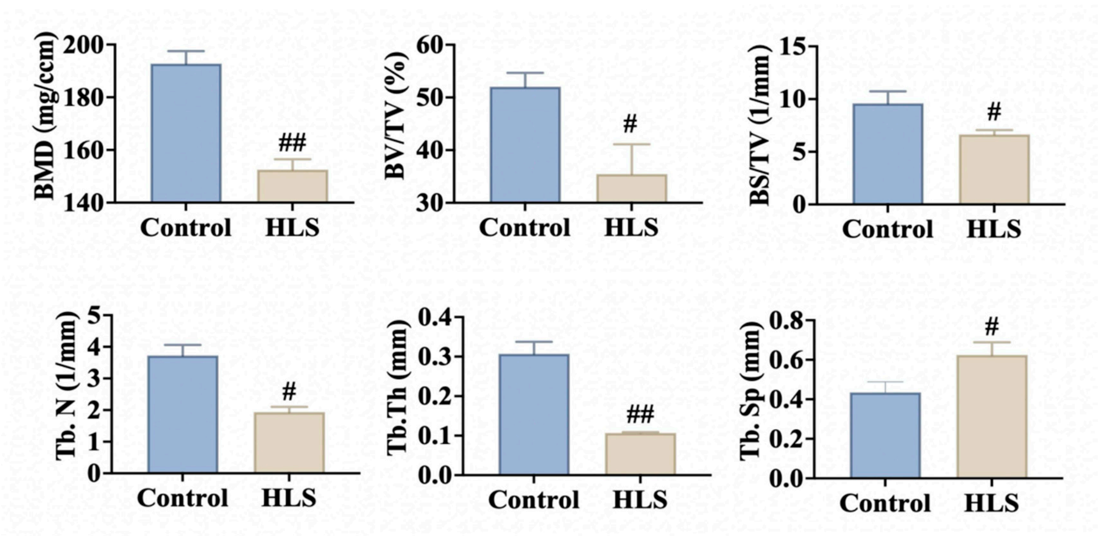

Figure S2. Statistical analysis of Micro CT parameters. Quantitative analysis of trabecular bone parameters, including BMD, BV/TV, BS/TV, Tb. N, Tb. Th and Tb.Sp in the distal femurs. Control:

untreated normal rats; HLS: hindlimb-suspended rats. Data are presented as mean ± SD (n = 6 per group). Statistical analysis was performed using unpaired Student's t-test. # $p < 0.05$, ## $p < 0.01$ vs. Control group;. Abbreviations: BMD: bone mineral density; BV/TV: bone volume fraction; BS/TV: bone surface density; Tb. Th, trabecular thickness; Tb. N, trabecular number; Tb. Sp, trabecular separation.

1.3 Effect of AMP1-1 on body weight growth and organ index.

As shown in Figure S3A, the HLS group exhibited a significant reduction in body weight gain rate compared to the Control group, while AMP1-1 treatment significantly restored the body weight gain rate. Importantly, as presented in Figure S3B, AMP1-1 treatment did not cause any significant alterations in the organ coefficients of the heart, liver, spleen, or kidney, suggesting that AMP1-1 has no obvious toxic effects on these major organs under the current experimental conditions.

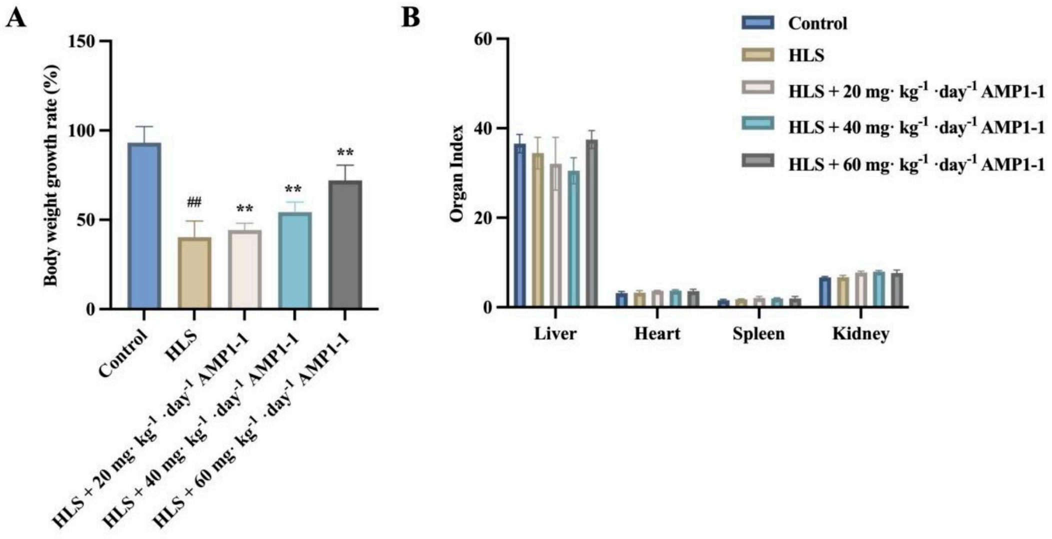

Figure S3. Effect of AMP1-1 on body weight growth and organ index. (A) Body weight growth rate of rats in each group over the experimental period. (B) Organ index (organ weight/body weight ratio) of the liver, heart, spleen, and kidney. Control: untreated normal rats; HLS: hindlimb-suspended rats; HLS+20, HLS+40, HLS+60: HLS rats treated with AMP1-1 at 20, 40, or 60 $mg \cdot kg^{-1} \cdot day^{-1}$, respectively. Data are presented as mean ± SD (n = 6 per group). Statistical analysis was performed using one-way ANOVA followed by Tukey's post-hoc test. ## $p < 0.01$ vs. Control group; ** $p < 0.01$ vs. HLS group.

Table S1. The information of differential expressed proteins in response to oxidative stress

Gene name	GO ID	Fold Change	*P*-value	Uniprot website
Mb	Q9QZ76	5.866	2.67E-10	http://www.uniprot.org/uniprot/Q9QZ76
Cryab	P23928	2.733	1.15E-20	http://www.uniprot.org/uniprot/P23928
Ppid	Q6DGG0	2.038	1.13E-12	http://www.uniprot.org/uniprot/Q6DGG0
Hsph1	Q66HA8	1.708	1.66E-17	http://www.uniprot.org/uniprot/Q66HA8
Gpx7	D3ZQI1	1.560	2.21E-23	http://www.uniprot.org/uniprot/D3ZQI1
Cat	P04762	0.648	2.98E-11	http://www.uniprot.org/uniprot/P04762
Lcn2	P30152	0.603	2.98E-11	http://www.uniprot.org/uniprot/P30152
Col1a1	P02454	0.584	1.25E-17	http://www.uniprot.org/uniprot/P02454
Anxa1	P07150	0.550	6.00E-10	http://www.uniprot.org/uniprot/P07150
Psmb5	G3V7Q6	0.532	2.98E-11	http://www.uniprot.org/uniprot/G3V7Q6
Cfh	G3V9R2	0.502	2.98E-11	http://www.uniprot.org/uniprot/G3V9R2
Msra	Q923M1	0.4806	2.98E-11	http://www.uniprot.org/uniprot/Q923M1

Table S2. Effects of AMP1-1 on bone biomechanical parameters in femurs as assessed by three-point bending

Parameters	Control	HLS	HLS+20	HLS+40	HLS+60
Max-Stress (Mpa)	176.4±17.78	153.5±24.20 $^{\#}$	163.0±20.28	177.1±20.37*	189.3±38.47*
Young's modulus (Mpa)	9069±1588	4099±1702 $^{\#\#}$	7401±1224**	7891±1581**	8221±1554**

Parameters	Control	HLS	HLS+20	HLS+40	HLS+60
Max-Load (N)	150.7±13.47	93.82±6.26$^{##}$	101.1±7.40*	105.6±8.83**	107.4±9.57**
Energy (J)	0.06095± 0.0098	0.03569± 0.0073 $^{##}$	0.04086± 0.0086	0.04645± 0.0094**	0.05143± 0.0098**
Maximum strain (%)	5.866±1.49	3.721±1.05$^{##}$	4.225±0.93	5.341±1.63*	5.848±1.59**
Failure Stress (Mpa)	167.2±33.82	171.0±28.84	162.9±23.34	169.3±40.22	175.9±42.58
Failure Load (N)	145.5±16.15	97.59±11.15$^{##}$	92.37±4.18	96.82±16.98	104.0±11.20
Failure strain (%)	5.847±1.50	4.131±0.95$^{#}$	5.269±1.66	5.672±1.73	4.361±0.86

Table S3. Primer sequences used for qRT-PCR analysis

Gene name		Primer Sequence (5'-3')
Runt-related transcription factor 2 (*Runx2*)	Forward	GTGCGGTGCAAACTTTCTCC
	Reverse	AATGACTCGGTTGGTCTCGG
Osterix (*OSX*)	Forward	AAAAGGAGGCACAAAGAAGC
	Reverse	GGTGGGTAGTCATTGGCATAG
Bone alkaline phosphatase (*BALP*)	Forward	CGACAGCAAGCCCAAGAG
	Reverse	CGCCCGTGTTGTGGTGTA
Cathepsin K (*Ctsk*)	Forward	CTCGGCGTTTAATTTGGGAGA
	Reverse	TCGAGAGGGAGGTATTCTGAGT

Gene name	Primer Sequence (5'-3')	
Nuclear factor of activated T-cells c1 (*NFATc1*)	Forward	GGAGAGTCCGAGAATCGAGAT
	Reverse	TTGCAGCTAGGAAGTACGTCT
Tartrate-resistant acid phosphatase 5b (*TRAP 5b*)	Forward	GCTGGAAACCATGATCACCT
	Reverse	TTGAGCCAGGACAGCTGAGT
Catalase (*CAT*)	Forward	CCACAGTCGCTGGAGAGTCA
	Reverse	GTTTCCCACAAGGTCCCAGTT
Superoxide dismutase (*SOD*)	Forward	TCCACCACCCTTAGGGCTCA
	Reverse	AGCCACATTGCCCAGGTCTC
Glutathione peroxidase (*GSH-Px*)	Forward	CGGTTTCCCGTGCAATCAGT
	Reverse	ACACCGGGGACCAAATGATG
Nuclear factor E2-related factor 2 (*Nrf2*)	Forward	ACCTAAAGCACAGCCAACAC
	Reverse	CCTCTAATCGGCTTGAATGT
Heme oxygenase 1 (*HO-1*)	Forward	GTCCCAGGATTTGTCCGAGG
	Reverse	GGAGGCCATCACCAGCTTAAA
Glyceraldehyde-3-phosphate dehydrogenase (*GAPDH*)	Forward	GAGCCCTTCCACAATGCCAAA
	Reverse	GTCGTGGAGTCTACTGGTGTC
